# Associations Between Motor Competence and Executive Functions in Children and Adolescents: A Systematic Review and Meta-analysis

**DOI:** 10.1007/s40279-024-02040-1

**Published:** 2024-05-20

**Authors:** Ran Bao, Levi Wade, Angus A. Leahy, Katherine B. Owen, Charles H. Hillman, Timo Jaakkola, David Revalds Lubans

**Affiliations:** 1 Centre for Active Living and Learning, University of Newcastle, Callaghan, NSW Australia; 2 College of Human and Social Futures, School of Education, University of Newcastle, Callaghan, NSW Australia; 3 Active Living Research Program, Hunter Medical Research Institute, New Lambton Heights, NSW, Australia; 4 SPRINTER, Prevention Research Collaboration, Sydney School of Public Health, The University of Sydney, Sydney, NSW Australia; 5 Center for Cognitive and Brain Health, Department of Psychology, Northeastern University, Boston, MA USA; 6 Department of Physical Therapy, Movement, and Rehabilitation Sciences, Northeastern University, Boston, MA USA; 7 Faculty of Sport and Health Sciences, University of Jyväskylä, Jyvaskyla, Finland

## Abstract

**Background:**

Motor competence and executive functions co-develop throughout childhood and adolescence, and there is emerging evidence that improvements in motor competence may have cognitive benefits in these populations. There is a need to provide a quantitative synthesis of the cross-sectional, longitudinal and experimental studies that have examined the association between motor competence and executive functions in school-aged youth.

**Objectives:**

The primary aim of our systematic review was to synthesise evidence of the association between motor competence and executive functions in school-aged children and adolescents (5–18 years). Our secondary aim was to examine key moderators of this association.

**Methods:**

We searched the PubMed, PsycINFO, Scopus, Ovid MEDLINE, SPORTDiscus and EMBASE databases from inception up to 27 June 2023. We included cross-sectional, longitudinal and experimental studies that assessed the association between motor competence (e.g., general motor competence, locomotor skills, object control skills and stability skills) and executive functions (e.g., general executive functions, inhibition, working memory and cognitive flexibility) in children and adolescents aged 5–18 years.

**Results:**

In total, 12,117 records were screened for eligibility, and 44 studies were included. From the 44 included studies, we meta-analysed 37 studies with 251 effect sizes using a structural equation modelling approach in the statistical program R. We found a small positive association (r = 0.18, [95% confidence interval (CI) 0.13–0.22]) between motor competence and executive functions. The positive associations were observed in cross-sectional (r = 0.17, [95% CI 0.13–0.22]), longitudinal (*r* = 0.15, [95% CI 0.03–0.28]) and experimental studies (*r* = 0.25, [95% CI 0.01–0.45]). We also found that general motor competence (*r* = 0.25, [95% CI 0.18–0.33]), locomotor (*r* = 0.15, [95% CI 0.09–0.21]), object control (*r* = 0.14, [95% CI 0.08–0.20]) and stability (*r* = 0.14, [95% CI 0.08–0.20]) skills were associated with executive functions. We did not find any moderating effects for participants’ age on the associations between motor competence and executive functions.

**Conclusions:**

Our findings suggest a small-to-moderate positive association between motor competence and executive functions in children and adolescents. The small number of experimental studies included in this review support the assertion that interventions targeting children’s motor competence may be a promising strategy to improve their executive functions; however, more research is needed to confirm these findings. Future studies should explore the underlying mechanisms linking motor competence and executive functions as their comprehension may be used to optimise future intervention design and delivery.

**PROSPERO Registration:**

CRD42021285134.

**Supplementary Information:**

The online version contains supplementary material available at 10.1007/s40279-024-02040-1.

## Key Points

**Table:** 

Findings from our systematic review and meta-analyses suggest a small-to-moderate positive association between motor competence and executive functions in children and adolescents.
Locomotor, object control and stability skills were all positively associated with executive functions (e.g., inhibition, working memory, cognitive flexibility).
Most observational studies focused on children, while a limited number of studies involved adolescent participants.
Due to the small number of experimental studies, we were unable to establish a causal link between motor competence and executive functions in young people.

## Introduction

Motor competence is defined as the mastery of physical skills and movement patterns that enable participation in a range of physical activities [1]. It includes locomotor (e.g., running and jumping), object control (e.g., catching and throwing) and stability (e.g., balancing and twisting) skills [1]. The umbrella term “motor competence” is often used interchangeably with “motor proficiency”, “motor performance”, “motor ability” and “motor coordination” [2]. The development of motor competence is associated with a range of psychological (e.g., perceived competence [3]), physiological (e.g., physical fitness [4, 5], healthy weight status [2, 6]), and behavioural (e.g., physical activity [6, 7]) benefits [8]. Further, there is accumulating evidence that developing motor competence may also have benefits for young people’s executive functions [9–11].

Executive functions (EFs) are complex and can be understood from multiple perspectives. For example, prior research has explored EFs from evolutionary (e.g., ability to make decisions and behave in a purposeful, goal-directed, future-oriented manner), syndrome-based (i.e., impairments in individuals’ cognitive functioning), neurobiological (i.e., changes in brain structure and function) and statistical (e.g., psychometric properties of tests that assess inhibitory control, updating/working memory and shifting/cognitive flexibility) perspectives [12, 13]. For the purposes of our review, we define EFs as higher-order cognitive processes underlying the selection, scheduling, coordination and monitoring of complex, goal-directed processes [14]. In this definition, EFs typically include three core components: inhibitory control (e.g., the ability to maintain focus and suppress prepotent or automated responses), updating/working memory (e.g., retaining and updating of information), and shifting/cognitive flexibility (e.g., the ability to shift attention to changing task demands) [14, 15].

Previous studies have found positive associations between executive functions and academic performance (e.g., mathematics achievement) [16–18], and between executive functions and quality of life in children and adolescents [19]. Alternatively, poor executive functions are related to mental health disorders (e.g., depression, conduct disorder, attention deficit hyperactivity) [20, 21], poor physical health (e.g., obesity) [22], as well as social problems (e.g., violence, crime, reckless behaviour) [23, 24] in children and adolescents [25]. Engagement in physical activity has been shown to benefit executive functions [26], and it has been hypothesised that this may occur via a range of neurobiological, psychosocial and behavioural mechanisms [27, 28]. There is growing interest in the idea that the development of motor competence might provide some explanation of the effects of physical activity on executive functions. Indeed, there is evidence that motor competence and executive functions may develop via similar pathways [29–31]. The same cortical and subcortical regions of the brain (comprising functional neural networks) are partially responsible for the development of motor competence and executive functions in childhood, including the prefrontal cortex, cerebellum and basal ganglia [30]. Consequently, just as there is a neurobiological basis for asserting that regular physical activity in children contributes to the development of executive functions, there is also a neurobiological justification for the belief that involvement in motor learning activities can similarly enhance executive functions [15]. As highlighted by Hill (2023) in their review on the relationship of motor competence to broader cognitive and social outcomes, physical activity may improve executive functions via its quantitative characteristics (e.g., intensity, frequency, etc.,) or via qualitative characteristics (e.g., motor skill complexity). Learning motor skills requires the input of executive processes of inhibition, working memory and cognitive flexibility [10], meaning that greater executive functioning could plausibly translate into the performance of complex or novel skills. In sum, there is likely a bidirectional relationship between executive functions and motor skills acquisition and performance.

A recent systematic review including a meta-analysis found a positive association between motor skills (except for object control skills) and executive functions in children [11]. However, this review only included children aged 3–12 years and did not examine the role of key moderators (e.g., study design). More recently, Hill and colleagues published a conceptual model and systematic review that included a qualitative synthesis of the association between motor competence and cognitive outcomes in young people [32]. Consistent with the model proposed by Lubans and colleagues [33], the authors acknowledged that motor competence may support cognitive development via a range of neurobiological, psychosocial and/or behavioural mechanisms, and pointed out that clear patterns of domain-specific relationships are lacking [32]. As noted by the authors, young people typically perform motor skills in environments (e.g., physical education and sport practice) that challenge both their motor and cognitive abilities, and thus likely contribute to improvements in both motor and cognitive development simultaneously. However, due to the small number of experimental studies, the authors were unable to draw conclusions regarding the causal relationship between motor competence and cognitive outcomes [32]. In their narrative review of the literature, Willoughby and Hudson [15] discuss the contribution of motor skills and physical activity to the development of executive functions in early childhood. Based on their review of the literature, they note that it remains unclear whether increases in physical activity are enough to explain improvements in executive functioning, and suggest that the development of children’s motor skills is more strongly associated with executive function development in early childhood than the frequency, duration or intensity of their physical activity.

Despite increasing interest in the link between motor competence and executive functioning, no previous systematic review has conducted a quantitative synthesis of this relationship in children and adolescents. Narrative syntheses of quantitative data have notable limitations, including lack of transparency and conclusions based on subjective interpretation [34, 35]. Alternatively, meta-analyses are considered a superior approach to data synthesis because they: (i) involve the calculation of effect sizes, (ii) can correct for small-scale studies that are not adequately powered, and (iii) allow for the examination of moderator effects. Therefore, the primary aim of our systematic review and meta-analysis was to assess the strength of the association between motor competence and executive functions in school-aged children and adolescents (5–18 years). Our secondary aim was to examine key moderators of this association.

## Methods

### Protocol and Registration

Our systematic review was conducted according to the 2020 Preferred Reporting Items for Systematic Reviews and Meta-Analyses (PRISMA) statement [36]. The current review was prospectively registered with PROSPERO (CRD42021285134).

### Identification of Studies and Search Strategy

We conducted a comprehensive literature search to identify studies on the associations between motor competence and executive functions in children and adolescents. We searched six electronic databases (PubMed, PsycINFO, Scopus, Ovid MEDLINE, SPORTDiscus and EMBASE) from the year of their inception up to 27 June 2023. After automatically and manually removing duplicates, the remaining studies were subjected to a screening process. Our search strategy was modified in accordance with each of the databases (e.g., using MeSH terms where possible), and included the following keywords: motor competence (e.g., locomotor, object control and stability skills), executive functions (e.g., inhibition, working memory, cognitive flexibility), and population (e.g., children, adolescents). The search terms are detailed in the Online Supplementary Material (OSM; Table S1).

Where possible, search results were limited by language (English), species (human) and type (journal). The reference lists of included studies were checked for additional relevant studies.

### Inclusion/Exclusion Criteria

Two authors independently screened titles, abstracts and full-texts (RB and LW) against inclusion and exclusion criteria. Any disagreements between the two authors were resolved via discussion, and a consensus was reached with two other authors (AAL and DRL). The following inclusion criteria were applied: (1) experimental or quasi-experimental studies designed to promote motor competency and executive functions (i.e., inhibition, working memory, and cognitive flexibility) in children and adolescents; (2) observational studies focused on the associations between motor competence and executive functions; (3) data on school-aged children and adolescents (5–18 years of age); (4) data on participants’ motor competency (i.e., motor proficiency, motor performance, fundamental movement/motor skills, motor ability and motor coordination) and executive function (i.e., inhibition, working memory, cognitive flexibility); (5) objective assessment such as process-based (e.g., Test of Gross Motor Development-3rd Edition (TGMD-3)) and/or product-based measures (e.g., Movement Assessment Battery for Children-2nd Edition (MABC-2)) of motor competence and performance-based measures of executive functions (e.g., flanker task, trail-making task, digit span task) were eligible for this review. The following exclusion criteria were applied: (1) only reported fine motor skills (e.g., writing); (2) utilised fitness as a proxy for motor competence (e.g., strength); (3) individuals living with disability (e.g., intellectual and/or physical disability); (4) measured perceived motor competence or used rating scales to measure executive functions; (5) reported general cognition, but did not provide outcome data specific to executive functions (either as a composite score or domain specific).

### Data Extraction

Information regarding study location, first author’s name, year of publication, design (i.e., experimental, quasi-experimental, longitudinal, or cross-sectional study design), participant age, sample size, measures of motor competence and executive functions, and correlations between motor competency and executive function outcomes, were independently extracted by two authors (RB and LW). Differences in the extraction and coding of information were discussed and resolved with two other authors (AAL and DRL). In accordance with previous studies [1, 3, 8], motor competence components were classified into three categories, including locomotor skills, object control skills, and stability skills. Additionally, those studies that only reported the association between the composite scores of motor competence (e.g., locomotor, object control, and stability skills) and executive functions were labelled as “general motor competence”. The term “motor competence” encompassed all motor competence components, including general motor competence, locomotor skills, object control skills, and stability skills. Similarly, executive function domains were also classified into three categories [25], including inhibition, working memory, and cognitive flexibility. Studies that only reported the association between motor competence and composite scores of multiple executive function domains (e.g., inhibition, working memory, and cognitive flexibility), were coded as “general executive functions”. The term “executive functions” comprised general executive functions, inhibition, working memory and cognitive flexibility. For tests of executive functions, where reported, we used information on the accuracy and reaction time (i.e., the reaction time of correct responses) for calculation of effect sizes. Where this was not reported, we used accuracy data, unless the primary outcome of the test was time to completion (e.g., in the trail-making test).

### Criteria for Risk of Bias Assessment

Risk of bias was evaluated independently by two authors (RB and LW). Experimental studies were assessed using the Cochrane Risk of Bias Tool (RoB 2.0) for randomised controlled trials (RCTs) and the Risk Of Bias In Non-randomised Studies-of Interventions (ROBINS-I) for non-randomised controlled trials [37]. Disagreements between assessors were resolved by discussion. The criteria were: (1) randomisation process; (2) deviations from intended interventions; (3) missing outcome data; (4) measurement of the outcomes; and (5) selection of the reported results. Observational studies were assessed using the following items developed from the Joanna Briggs Institute critical appraisal checklists [37]: (1) study design allowed for causal inference (only for longitudinal study design); (2) random selection of study participants and/or study sites; (3) detailed description of sample characteristics; (4) valid assessment of motor competence; (5) valid assessment of executive functions; and (6) adjustment for relevant confounders in the analysis. Based on the assessment criteria, the risk bias for each included study was categorised based on the percentage of items rated as “yes” > 70% (low), 50–69% (moderate), or < 50% (high) [38, 39].

### Meta-analyses

We combined effect sizes using a structural equation modelling approach to multilevel meta-analysis. The main advantage of this approach is that it is not limited by the assumption of independence (i.e., effect sizes are nested within studies), and multiple effect sizes can be included from each study [40]. Unconditional mixed-effects models using maximum likelihood estimation were conducted to calculate the overall pooled effect size. For each pooled effect size, 95% likelihood-based confidence intervals (Cis) were calculated. All analyses were conducted using the metaSEM package in R Version 4.2.2 (code provided in OSM 1) [41].

We extracted various summary measures, including standardised mean differences and correlation coefficients. Summary metrics that were not reported as r values were converted to correlation coefficients (*r*) for observational and experimental studies. For observational studies, correlation coefficients were extracted according to whether the effect sizes were cross-sectional effect (one point in time) or longitudinal (changes over time). Correlation coefficients that controlled for relevant covariates (e.g., age and sex) were extracted. Where these were not available, the zero-order correlation coefficients were extracted. The correlation coefficients were converted into Fisher’s z for all analyses using the following formula (Eq. 1) [42]:

1 $$z = 0.5*\ln \left( {\frac{1 + r}{{1 - r}}} \right),$$

where ln is the natural logarithm [43]. Then, the results were converted back to r-values using the following conversion formulas (Eq. 2):

2 $$r = \frac{{\left( {e^{2*z} - 1} \right)}}{{\left( {e^{2*z} + 1} \right)}},$$

where *e* refers to the base of the natural logarithm [44]. For experimental studies, correlation coefficients (*r*) of change scores for both motor competence and executive functions between pre and post measurements were extracted from one study. For another study, we used the pre and post experimental means and standard deviations to calculate Cohen’s *d* using the formula (Eq. 3):

3 $$d = \frac{{\overline{\rm X}_{1} - \overline{\rm X}_{2} }}{{s_{d} }},$$

where ${\overline{\mathrm{\rm X}} }_{1}$ is the mean change in the first group, ${\overline{\mathrm{\rm X}} }_{2}$ is the mean change in the second group, ${s}_{d}$ is the pooled standard deviation of the two groups [42]. Then, Cohen’s *d* was converted into r values using the following formula (Eq. 4):

4 $$r = \frac{d}{{\sqrt {d^{2} + 4} }},$$

where $d$ is Cohen’s *d* [42]. To aid with interpretation, the correlation coefficients are referred to as small (0.10–0.20), moderate (0.21–0.35) or large (> 0.35) [45]. For consistency, negative summary outcomes (i.e., where negative numbers indicate greater performance – such as outcomes including reaction time) were inverted to positive summary measures. In addition, 95% likelihood-based CIs were calculated for each effect size.

Heterogeneity was assessed using the *I*^2^ statistic, with values from 0–40%, 30–60%, 50–90%, and 75–100% indicating low, moderate, substantial, and high heterogeneity, respectively [46]. Publication bias was analysed using funnel plots and Egger’s regression test, which provides a metric of the asymmetry present in the plot (with a *p*-value > 0.05 indicating no evidence of publication bias). Moderator analyses were conducted to determine whether the association between motor competence and executive functions differed according to the type of motor competence (e.g., locomotor, object control, and stability skills), executive function domain (e.g., inhibition, working memory, cognitive flexibility), age of participants (treated as a continuous variable), and study design (e.g., cross-sectional, longitudinal, experimental). Initially, we treated age as a binary moderator, but age did not influence the association between motor competence and executive functions. Given the small number of the included studies involving adolescents, we subsequently treated age as a continuous moderator, which allowed for a more precise examination of its effects on the association between motor competence and executive functions. The R^2^ values were computed to determine the proportion of explained variance for each potential moderator variable.

## Results

### Study Selection

**Fig. 1:**
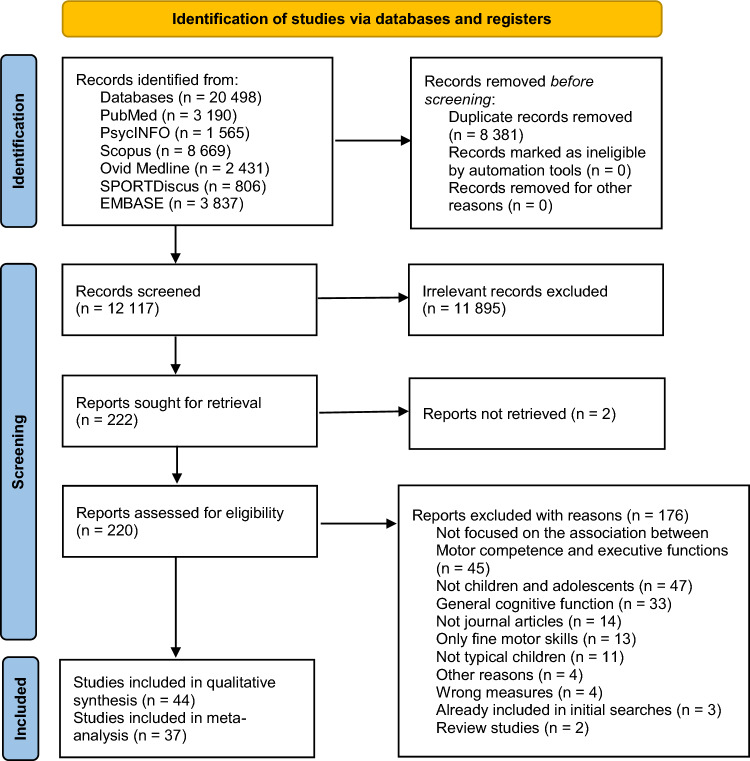
PRISMA flow diagram for searching and selection of the included studies

After conducting a comprehensive literature search, a total of 20,498 records were identified. After removing 8,381 duplicates, 12,117 records were screened via title and abstract for suitability. After the removal of irrelevant records, 220 records were assessed for eligibility via full-text screening. Finally, 44 studies were included in the qualitative synthesis and 37 studies [47–83] were included in the meta-analysis (Fig. 1).

### Characteristics of Included Studies

Study characteristics are presented in Table S2 (OSM). Most studies were conducted in Germany (*n* = 7) [49, 55, 61, 64, 70, 73, 74], followed by Switzerland (*n* = 6) [53, 54, 56, 62, 75, 76], the Netherlands (*n* = 6) [63, 69, 78, 84–86], Australia (*n* = 5) [47, 52, 57, 66, 87], Brazil (*n* = 3) [48, 50, 88], Italy (*n* = 3) [51, 65, 89], the USA (*n* = 2) [58, 72], Norway (*n* = 2) [71, 79], China (*n* = 2) [77, 81], Denmark (*n* = 1) [90], Finland (*n* = 1) [59], Greece (n = 1) [83], Ireland (*n* = 1) [82], Portugal (*n* = 1) [67], and Spain (*n* = 1) [80]. One study focused on children from Germany and Switzerland [68], and another targeted children from Germany and Oman [60]. Cross-sectional designs were used in 34 studies [48–50, 53–55, 57, 59, 60, 62–67, 69, 71, 73, 75–90], with the remainder consisting of longitudinal (*n* = 5) [47, 56, 61, 68, 74], randomised controlled trials (*n* = 3) [52, 58, 70] and non-randomised controlled trials (*n* = 2) [51, 72]. Most studies included children between the ages of 5 and 12 years (*n* = 39), with the remaining five studies focusing on adolescents (between the ages of 13 and 18 years) [53, 57, 59, 64, 87].

### Measurement of Motor Competence and Executive Functions

Locomotor skills were examined in 26 studies [47, 48, 50–54, 56, 58, 59, 62, 63, 67, 69, 72, 74–78, 81–84, 86, 88], object control skills were reported in 28 studies [48, 49, 52–55, 57–61, 63, 64, 66, 69–71, 74, 77, 78, 80, 82–87, 89], and stability skills were assessed in 32 studies [49, 50, 53–55, 57, 61–63, 65–70, 72–75, 78–90]. The most widely used measurement tools for assessing motor competence were the Movement Assessment Battery for Children (MABC) [49, 55, 57, 61, 66, 70–72, 78, 80, 87, 89] and the Körperkoordinationstest Für Kinder (KTK) [48, 50, 62–64, 67, 69, 75, 81, 84, 86, 88], which were both used in 12 studies. Additionally, six studies used the Bruininks-Oseretsky Test of Motor Proficiency, second edition (BOT-2) [63, 69, 73, 82, 83, 86], three studies used the Basic Motor Competence in Fifth Grades (MOBAK-5) [53, 54, 74], and the Test of Gross Motor Development, second edition (TGMD-2) was used in four studies [48, 58, 77, 82]. Additionally, one study used the Peabody Developmental Motor Scale-Second Edition (PDMS-2) to assess motor competence [51]. Three core components of executive functions were measured, including inhibition (*n* = 21) [51, 52, 54, 56, 57, 59, 61–63, 66, 70–73, 75, 78, 80, 81, 84, 86, 89], working memory (*n* = 33) [47, 51–57, 59–64, 68, 69, 71, 73–75, 77–87, 89, 90], and cognitive flexibility (*n* = 17) [50–52, 54, 57, 62, 64, 70–73, 75, 76, 80, 81, 83, 88]. The most common tests used to assess inhibition were flanker tasks (*n* = 15) [48, 52, 54, 56, 59, 61–63, 65, 72, 73, 75, 76, 80, 81] and stop signal tasks [63, 66, 78, 84, 86]. Cognitive flexibility was most often assessed using the mixed flanker task [54, 56, 62, 72], the trail-making task [50, 51, 71, 88], and the dimensional change card sort test [52, 75, 80]. The digit span test [55, 60, 63, 64, 71, 78, 83, 84, 86, 91] and N-back tasks [47, 54, 56, 57, 81, 87] were the most common measures of working memory. A list of the executive function measures used in the included studies can be found in Table S3 (OSM).

### Risk of Bias of Included Studies

Based on the information provided, 65.9% of the included studies were considered to have a low risk of bias, while the remaining studies had a moderate risk (all risk-of-bias information is in OSM 2). Among cross-sectional studies, the majority (70.6%) were considered to have a low risk of bias, with 29.4% studies having a moderate risk of bias. Regarding the measurement tools of all studies, a high percentage of studies used valid and reliable instruments to assess motor competence (91.2%) and executive functions (76.5%). Additionally, 61.8% of included studies controlled for relevant covariates when examining the association between motor competence and executive functions. Among longitudinal studies, 60% were considered to have a low risk of bias and the remaining studies had a moderate risk of bias. All longitudinal studies used valid and reliable measurement tools to assess motor competence, and the majority (80%) used valid and reliable measurement tools to assess executive functions. All longitudinal studies controlled for relevant covariates when examining the association between motor competence and executive functions. All RCTs were deemed to have a moderate risk based on the following criteria: “bias due to deviations from intended intervention” and “bias in selection of the reported result”. Both quasi-experimental studies were considered to have a low risk of bias.

### Synthesis of Results

#### Overall Associations

**Fig. 2:**
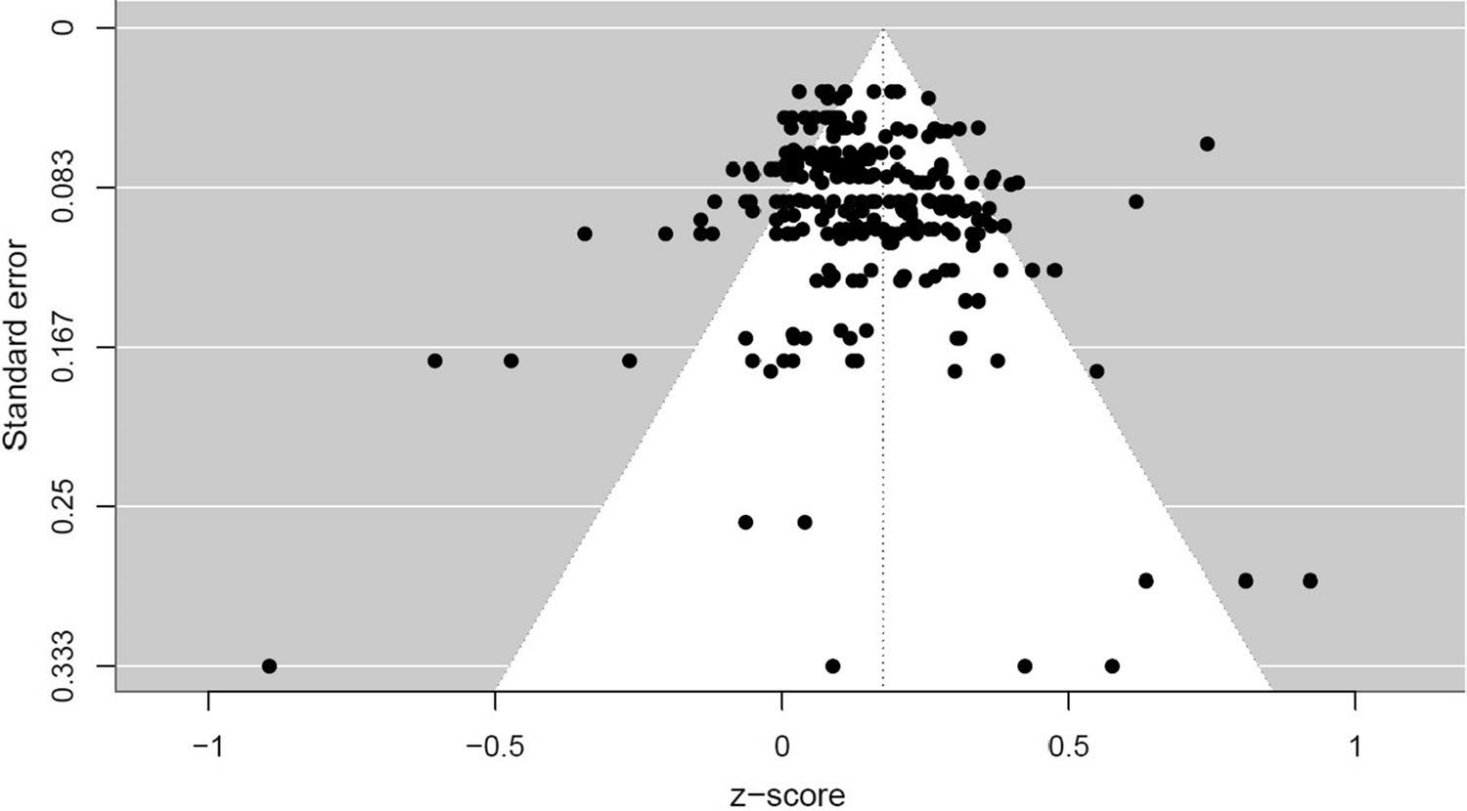
The funnel plot (Fisher’s* z*) for all the included studies in this meta-analysis showed no evidence of publication bias

Table 1 displays the results of the meta-analysis of the association between motor competence and executive functions. Overall, the findings of the meta-analysis, which included 37 studies and 251 effect sizes, indicate a small positive association between motor competence and executive functions in children and adolescents (*r* = 0.18, 95% CI 0.13–0.22). For this effect size, there was low heterogeneity within studies (*I*^2^ = 0.15) and moderate heterogeneity between studies (*I*^2^ = 0.63). The funnel plot (Fig. 2) and Egger’s regression (*z* = 1.18, *p* = 0.24) suggest that there was no evidence of publication bias.

**Table 1:** Associations between motor competence and executive functions in children and adolescents

Variable	No. of studies (No. of ES)	Pooled ES	95% CI	*Q*	*R*^2^_2	*R*^2^_3	Tau2_2	Tau2_3	*I*^2^_2	*I*^2^_3
Summary effect	37 (251)	0.18	0.13–0.22	*Q*_250_ = 709.70 (*p* = 0)			< 0.01	0.02	0.15	0.63
Moderation										
Design	37 (251)			*Q*_250_ = 709.70 (*p* = 0)	0.00	0.03	< 0.01	0.02		
Cross-sectional	34 (239)	0.17	0.13–0.22							
Longitudinal	4 (8)	0.15	0.03–0.28							
Experimental	2 (4)	0.25	0.01–0.45							
Age (treated as continuous)	36 (247)	< 0.01	– 0.03–0.04	*Q*_246_ = 697.65 (*p* = 0)	0.04	0.00	< 0.01	0.05		
Motor competence types	37 (251)			*Q*_250_ = 709.70 (*p* = 0)	0.15	0.00	< 0.01	0.02		
General	13 (41)	0.25	0.18–0.33							
Locomotor	12 (74)	0.15	0.09–0.21							
Object control	15 (64)	0.14	0.08–0.20							
Stability	17 (72)	0.14	0.08–0.20							
Executive function domains	37 (251)			*Q*_250_ = 709.70 (*p* = 0)	0.19	0.00	< 0.01	0.02		
General	5 (14)	0.29	0.21–0.36							
Inhibition	21 (74)	0.18	0.13–0.23							
Working Memory	28 (103)	0.17	0.12–0.23							
Cognitive Flexibility	17 (60)	0.13	0.08–0.19							

*EFs* executive functions, *Pooled ES* pooled effect sizes, *Heterogeneity at Level 2* ES from the same study, *Heterogeneity at Level 3* ES from the different studies, I^2^ is only available for the overall ES. Fisher’s z has been converted to r values

General motor competence (*r* = 0.25, 95% CI 0.18–0.33), locomotor (*r* = 0.15, 95% CI 0.09–0.21), object control (*r* = 0.14, 95% CI 0.08–0.20) and stability (*r* = 0.14, 95% CI 0.08–0.20) skills were all positively associated with executive functions. Motor competence components explained the small variance of the heterogeneity within (Tau^2^ < 0.01) and between (Tau^2^ = 0.02) studies, while demonstrating negligible variation within (*R*^2^ = 0.15) and between studies (*R*^2^ = 0). In terms of motor competence, positive associations were observed for general executive functions (*r* = 0.29, 95% CI 0.21–0.36), inhibition (r = 0.18, 95% CI 0.13–0.23), working memory (*r* = 0.17, 95% CI 0.12–0.23), and cognitive flexibility (*r* = 0.13, 95% CI 0.08–0.19). Executive function domains explained little variance in the heterogeneity within (Tau^2 ^ < 0.01) and between (Tau^2^ = 0.02) studies, while demonstrating negligible variation within (*R*^2^ = 0.19) and between studies (*R*^2^ = 0). Regarding the role of study design, positive associations were found for cross-sectional (*r* = 0.17, 95% CI 0.13–0.22), longitudinal (*r* = 0.15, 95% CI 0.03–0.28) and experimental (one RCT and one quasi-experimental study) (*r* = 0.25, 95% CI 0.01–0.45) studies. Study design explained none of the heterogeneity within (*R*^2^ = 0) and little between (*R*^2^ = 0.03) studies. Age was not a moderator of the association between motor competence and executive functions (*r* < 0.01, 95% CI – 0.03 to 0.04).

#### Motor Competence and Executive Function Domains

*Motor competence and general executive functions:* The results pertaining to general executive functions are outlined in Table 2. A positive association was observed between motor competence and general executive functions (*r* = 0.29, 95% CI 0.22–0.35). Most of the heterogeneity was attributed to the differences observed within studies (*I*^2^ = 0.57) and no heterogeneity was observed between (*I*^2^ = 0) studies. General motor competence (*r* = 0.34, 95% CI 0.25–0.42), locomotor (*r* = 0.32, 95% CI 0.18–0.44), object control (*r* = 0.23, 95% CI 0.13–0.33), and stability (*r* = 0.19, 95% CI 0.02–0.36) skills were positively associated with general executive functions. Motor competence components explained a small proportion of the heterogeneity within studies (*R*^2^ = 0.32) and negligible variation between studies (*R*^2^ = 0). No effect of age (*r* = – 0.01, 95% CI – 0.03 to 0.11) was observed in the association between motor competence and general executive functions.

**Table 2:** Associations between motor competence and general executive functions in children and adolescents

Variable	No. of studies (no. of ES)	Pooled ES	95% CI	Q	R^2^_2	R^2^_3	Tau2_2	Tau2_3	I^2^_2	I^2^_3
Summary effect	5 (14)	0.29^a^	0.22^a^–0.35^a^	Q_13_ = 30.61 (p = 0.00)			0.01	< 0.01	0.57	0.00
Moderation										
Age (treated as continuous)	5 (14)	-0.01	-0.13–0.11	Q_13_ = 30.61 (p = 0.00)	0.00	0.00	0.02	0.08		
Motor competence types				Q_13_ = 30.61 (p = 0.00)	0.32	0.00	0.01	< 0.01		
General	3 (6)	0.34^a^	0.25^a^–0.42^a^							
Locomotor	1 (3)	0.32^a^	0.18^a^–0.44^a^							
Object control	2 (3)	0.23^a^	0.13^a^–0.33^a^							
Stability	2 (2)	0.19^a^	0.02^a^–0.36^a^							

^a^Pooled effect sizes calculated using Wald's confidence intervals (CIs)

*Motor competence and inhibition:* For inhibition (Table 3), there was a positive association with motor competence (*r* = 0.15, 95% CI 0.11–0.18). Low heterogeneity was attributed to the differences observed within (*I*^2^ = 0.07) and between (*I*^2^ = 0.32) studies. General motor competence (*r* = 0.12, 95% CI 0.03–0.20), locomotor (*r* = 0.14, 95% CI 0.09–0.18), object control (*r* = 0.16, 95% CI 0.11–0.21), and stability (*r* = 0.16, 95% CI 0.12–0.21) skills were positively associated with inhibition. Motor competence components explained a large proportion of the heterogeneity within studies (*R*^2^ = 0.65) and negligible variation between studies (*R*^2^ = 0). The positive association between motor competence and inhibition was observed in cross-sectional (*r* = 0.14, 95% CI 0.11–0.18) and experimental (*r* = 0.24, 95% CI 0.05–0.41) studies. However, no positive association was observed in only one longitudinal study (*r* = 0.15, 95% CI – 0.03 to 0.31). Study design explained a small proportion of the heterogeneity within studies (*R*^2^ = 0.01) and negligible variation between studies (*R*^2^ = 0.03). No effect of age (*r* =  0.01, 95% CI – 0.02 to 0.03) was observed in the association between motor competence and inhibition.

**Table 3:** Associations between motor competence and inhibition in children and adolescents

Variable	No. of studies (no. of ES)	Pooled ES	95% CI	*Q*	*R*^2^_2	*R*^2^_3	Tau2_2	Tau2_3	*I*^2^_2	*I*^2^_3
Summary effect	21 (74)	0.15	0.11–0.18	*Q*_73_ = 129.40 (*p* < 0.001)			< 0.01	< 0.01	0.07	0.32
Moderation										
Design	21 (74)			*Q*_73_ = 129.40 (*p* < 0.001)	0.01	0.03	< 0.01	< 0.01		
Cross-sectional	18 (71)	0.14	0.11–0.18							
Longitudinal	1 (1)	0.15	– 0.03–0.31							
Experimental	2 (2)	0.24	0.05–0.41							
Age (treated as continuous)	21 (74)	0.01	– 0.02–0.03	*Q*_73_ = 129.40 (*p* < 0.001)	0.05	0.00	< 0.01	0.03		
Motor competence types	21 (74)									
General	5 (8)	0.12	0.03–0.20	*Q*_73_ = 129.40 (*p* < 0.001)	0.65	0.00	< 0.01	< 0.01		
Locomotor	7 (23)	0.14	0.09–0.18							
Object control	8 (18)	0.16	0.11–0.21							
Stability	12 (25)	0.16	0.12–0.21							

*Motor competence and working memory:* We observed a positive association between motor competence (*r* = 0.16, 95% CI 0.12–0.20) and working memory (Table 4). Low heterogeneity was attributed to differences observed within (*I*^2^ = 0.19) and moderate heterogeneity between (*I*^2^ = 0.41) studies. General motor competence (*r* = 0.20, 95% CI 0.13–0.28), locomotor (*r* = 0.14, 95% CI 0.09–0.20), object control (*r* = 0.14, 95% CI 0.08–0.19) and stability (*r* = 0.15, 95% CI 0.09–0.20) skills were positively associated with working memory. Motor competence components explained a small proportion of the heterogeneity within (R^2^ = 0.20) and between (*R*^2^ = 0.13) studies. Positive associations between motor competence and working memory were observed in cross-sectional (*r* = 0.16, 95% CI 0.12–0.20) and longitudinal (*r* = 0.14, 95% CI 0.03–0.26) studies. Study design explained a small proportion of the heterogeneity within (*R*^2^ = 0) and between (*R*^2^ = 0.01) studies. No effect of age (*r* < 0.01, 95% CI – 0.03 to 0.04) was observed in the association between motor competence and working memory.

**Table 4:** Associations between motor competence and working memory in children and adolescents

Variable	No. of studies (no. of ES)	Pooled ES	95% CI	*Q*	*R*^2^_2	*R*^2^_3	Tau2_2	Tau2_3	*I*^2^_2	*I*^2^_3
Summary effect	28 (103)	0.16	0.12–0.20	Q_102_ = 226.30 (p < 0.001)			< 0.01	0.01	0.19	0.41
Moderation										
Design	28 (103)			Q_102_ = 226.30 (p < 0.001)	0.00	0.01	< 0.01	0.01		
Cross-sectional	26 (96)	0.16	0.12–0.20							
Longitudinal	4 (7)	0.14	0.03–0.26							
Age (treated as continuous)	28 (103)	< 0.01	– 0.03–0.04	Q_102_ = 226.30 (p < 0.001)	0.00	0.00	< 0.01	0.04		
Motor competence types	28 (103)			Q_102_ = 226.30 (p < 0.001)	0.20	0.13	< 0.01	< 0.01		
General	9 (21)	0.20	0.13–0.28							
Locomotor	10 (26)	0.14	0.09–0.20							
Object control	12 (30)	0.14	0.08–0.19							
Stability	11 (26)	0.15	0.09–0.20							

*Motor competence and cognitive flexibility:* We found a positive association between motor competence (r = 0.14, 95% CI 0.03–0.25) and cognitive flexibility (Table 5). Most of the heterogeneity was attributed to differences observed between studies (*I*^2^ = 0.86). Locomotor (*r* = 0.20, 95% CI 0.08–0.30), object control (*r* = 0.14, 95% CI 0.02–0.26), and stability (*r* = 0.15, 95% CI 0.03–0.26) skills were positively associated with cognitive flexibility. Motor competence components explained a large proportion of heterogeneity within (*R*^2^ = 0.59) studies. We observed a positive association between motor competence and cognitive flexibility in cross-sectional studies (*r* = 0.13, 95% CI 0.02–0.24). However, no positive association was observed in only one experimental (*r* = 0.26, 95% CI – 0.22 to 0.64) study. No heterogeneity was found within (*R*^2^ = 0) and between (*R*^2^ = 0.01) studies. No effect of age (*r* = 0.01, 95% CI – 0.04 to 0.07) was observed in the association between motor competence and cognitive flexibility.

**Table 5:** Associations between motor competence and cognitive flexibility in children and adolescents

Variable	No. of studies (no. of ES)	Pooled ES	95% CI	*Q*	*R*^2^_2	*R*^2^_3	Tau2_2	Tau2_3	I^2^_2	I^2^_3
Summary effect	17 (60)	0.14	0.03–0.25	*Q*_59_ = 264.36 (p = 0)			< 0.01	0.04	0.01	0.86
Moderation										
Design	17 (60)			*Q*_59_ = 264.36 (*p* = 0)	0.00	0.01	< 0.01	0.04		
Cross-sectional	16 (58)	0.13	0.02–0.24							
Experimental	1 (2)	0.26	– 0.22–0.64							
Age (treated as continuous)	16 (56)	0.01	– 0.04–0.07	*Q*_55_ = 252.41 (*p* = 0)	0.24	0.00	< 0.01	0.07		
Motor competence types	17 (60)			*Q*_59_ = 264.36 (*p* = 0)	0.59	0.11	< 0.01	0.04		
General	3 (6)	< 0.01^a^	– 0.24^a^–0.25^a^							
Locomotor	7 (22)	0.20^a^	0.08^a^–0.30^a^							
Object control	6 (13)	0.14^a^	0.02^a^–0.26^a^							
Stability	9 (19)	0.15^a^	0.03^a^–0.26^a^							

^a^Pooled effect sizes calculated using Wald’s confidence intervals (CIs)

## Discussion

### Summary of Findings

This is the first systematic review and meta-analysis to comprehensively synthesise the associations between motor competence (e.g., locomotor skills, object control skills, stability skills) and executive functions (e.g., inhibition, working memory, cognitive flexibility) in children and adolescents. Our main findings suggest a small positive association between motor competence and executive functions in these populations that is consistent across cross-sectional, longitudinal and experimental studies. Our analyses revealed that all types of motor competence domains were significantly associated with each of the executive functioning domains. Similarly, age had no influence on the association between motor competence and executive functions, suggesting a consistent relationship between these outcomes across the ages included in the analysis.

### Motor Competence and Executive Functions

Our meta-analysis provides support for the hypothesis that motor competence and executive functions in children and adolescents are connected [10, 30]. Although the magnitude of effect size was small (*r* = 0.18, 95% CI 0.13–0.22), the associations were consistent across types of motor competence and domains of executive function. Inhibition, working memory and cognitive flexibility are all involved in skill acquisition, and it has been proposed that the allocation of cognitive resources during skill acquisition may contribute to improvements in executive functioning [10, 92, 93]. The current review includes evidence from experimental studies, the findings of which suggest that motor competence interventions may improve children’s executive functions [58, 72]. To illustrate, a 6-week motor competence intervention, targeting the improvement of locomotor and object control skills, demonstrated a positive relationship between the composite score of the two skills and enhancement in the composite score of executive functions [58]. In another intervention lasting 7 weeks, the results suggested the enhancement of balance was related to greater inhibition and cognitive flexibility in children [72]. However, our risk of bias identified a number of methodological concerns with these studies, including: (1) deviations from intended intervention (e.g., variations in delivery resources), and (2) bias in the selection and reporting of outcomes (e.g., not providing the information on trial registration, not reporting the association between specific motor competence components and executive function sub-domains) [52, 58, 70]. Additionally, only two experimental studies were included in this analysis, meaning these findings should be interpreted with caution. Despite these limitations, these findings are in accordance with previous research indicating that motor competence acquisition may influence several brain regions and their associated networks, such as the prefrontal cortex, cerebellum, and basal ganglia [30, 94].

Our moderator analyses indicate that age did not moderate the association between motor competence and executive functions. Based on the current evidence, there is no indication that the strength of the relationship between motor competence and executive functions significantly differs from childhood to adolescence. However, it should be noted that only five studies in our systematic review involved adolescents. Further study is needed to better understand the association between motor competence and executive functions across these developmental stages. Findings from the longitudinal research in this review provide some indication that motor competence may predict improved executive functions. However, there are some nuances of the findings that are worth noting. For instance, in a sample of 8-year-olds, Rigoli et al. [47] found that working memory performance was predictive of later motor competence, rather than the other way around. In their longitudinal investigation, Ludyga et al. [74] found no longitudinal relationship between motor competence and working memory performance. However, through analysis of electroencephalography, the authors found that children with low motor competency experience a change toward use of a less efficient cognitive control strategy (as indicated by a decrease in cue P300 and the initial contingent negative variation). This suggests that motor competence may not directly impact executive functions as measured by behavioural tasks. Instead, it may manifest through alterations in the underlying cognitive control mechanisms, such as changes in one’s approach to complex tasks. The findings also suggest that the inclusion of neurophysiological markers alongside behavioural tasks may help us to gain a comprehensive understanding of the relationship between motor skills and changes in executive function, and to identify potential mechanisms.

Our findings are generally consistent with previous reviews on this topic [9–11, 15]. In their review, Willoughby and Hudson [15] concluded that there is a weak to moderate association between motor competence and executive functions in early childhood [15]. A meta-analysis investigating the relationship between motor skills and executive functions found a small but positive association (r = 0.18, 95% CI 0.126–0.246) in children [11]. However, their further analyses found that only stability exhibited a positive association with all sub-domains of executive functions [11]. It is worth noting that this earlier meta-analysis incorporated data from preschool children aged 3–5 years and that differences in the relationship of motor competence to executive functions may exist between preschool children and school-aged children [1, 48, 95]. Moreover, the previous meta-analysis also included self-, teacher- and parent-reported ratings of children’s executive functions (i.e., the Behaviour Rating Inventory of Executive Function–parent version) [11]. Several concerns regarding rating scales should be acknowledged, including weak construct and content validity, as well as the influence of the characteristics of raters on their ratings (e.g., education levels, experience with similar rating scales) [96]. The current study exclusively included performance-based measures as they offer a more objective assessment of executive functions [96].

Most of the studies included in our review used product-oriented instruments (e.g., MABC-2, KTK, BOT-2, MOBAK-5) to assess motor competence, while process-oriented measures (e.g., TGMD-2, PDMS-2) were used in only five studies. It is widely acknowledged that product-oriented tests primarily focus on outcome measures, such as the number of tosses or the distance of throwing, whereas process-oriented assessments evaluate the quality (e.g., form, mechanics of movement) of motor competence [97, 98]. It appears that process-oriented (e.g., TGMD-2) and product-oriented (e.g., KTK, BOT-2) instruments evaluate distinct facets of motor competence. The scientific evidence indicates a moderate level of agreement (with a variance of 27%) between MABC-2 and TGMD-2 in assessing motor competence in 5- to 8-year-old children [99]. It is noteworthy that TGMD is widely employed to assess motor competence, yet it does not include any stability skills [48, 82]. As such, it is recommended to include further studies to provide a comprehensive evaluation of motor competence [97, 98]. Researchers also highlighted several concerns associated with using different measures of motor competence, including the sensitivity and discriminatory capabilities, translating “success” in skill performance [100]. Consequently, the absence of a universally accepted “gold standard” measure for assessing motor competence poses challenges in synthesising effect sizes across specific sub-domains. It potentially impedes the identification of associations between specific motor competence and their impact on health outcomes. In general, there is a need for “gold standard” measures to assess both motor competence and executive functions.

Despite a positive association between general motor competence and general executive functions, drawing definitive conclusions from these results is challenging. This difficulty arises because the included effect sizes are derived from studies that examined different combinations of executive functions. For instance, certain studies solely reported the composite score of executive functions that were not domain-specific (e.g., inhibition, working memory, cognitive flexibility) [48, 57, 71, 75]. Although a higher effect was found, the associations between specific components of motor competence and different executive function sub-domains may provide more insight into the nature of these relationships.

### Strengths and Limitations

To the best of our knowledge, this is the first systematic review to quantitatively synthesize the association between motor competence and executive functions in children and adolescents. A notable strength of our review is the multilevel structural equation modelling approach that adheres to best practice guidelines in sport and exercise science [101]. However, there are limitations that should be noted. First, it is important to acknowledge most of the studies used product-oriented instruments to assess motor competence. It is evident that process-oriented (e.g., TGMD-2) and product-oriented (e.g., MABC-2) instruments assess distinct facets of motor competence [97, 98]. Second, most of the included studies were observational, thus limiting our ability to infer causation. Third, due to the small number of studies involving adolescents, we were unable to draw firm conclusions regarding the relationship between motor competence and executive functions in this developmental stage. Fourth, using individual participant data (IPD) may be more suitable for our understanding of the association between motor competence and executive functions in children and adolescents. However, this approach requires a significant amount of work, as well as author and ethics approval from each institution. Finally, none of the included studies examined the underlying mechanisms of the association between motor competence and executive functions. Therefore, to gain a comprehensive understanding of this association in children and adolescents, further studies focused on the testing potential mechanisms are needed.

### Future Directions

Our findings suggest a positive association between motor competence and executive functions, which may be attributed to the presence of shared functional regions in the brain. Several studies have demonstrated that motor competence acquisition may impact various brain regions and their associated networks, including the prefrontal cortex, cerebellum and basal ganglia [30, 94]. The cerebellum plays a crucial role in instructing and guiding the prefrontal cortex to engage in “think ahead” functions, demonstrating the collaborative nature of these brain regions as an integrated network [93]. The functional regions of the brain are known to develop alongside executive functions in children and adolescents [102, 103]. Owing to a scarcity of evidence, the underlying mechanisms of this association remain unstudied. Therefore, further experimental studies are warranted to identify underlying mechanisms for this association. Additionally, motor competence is associated with physical activity and physical fitness [6, 15], both of which have benefits for executive functions in children and adolescents [104, 105]. Notably, there are indications that physical fitness may act as a moderator in the association between motor competence and executive functions [10]. However, none of the experimental studies in our systematic review considered physical activity or physical fitness when investigating this relationship. Given the interconnected association among physical activity, physical fitness, motor competence and executive functions [2, 10], more studies are needed in this area.

Our meta-analysis did not find any effect of age on the association between motor competence and executive functions. It is worth noting that most of the included studies were conducted in children (5–12 years of age), with only five studies involving adolescents (13–18 years of age). Given that both motor competence and executive functions continue to develop throughout adolescence, the relationship between the two may differ compared to childhood. Therefore, more research with adolescents may provide a more comprehensive perspective on how motor competence and executive functions are interconnected across development.

Researchers have proposed that motor tasks that are difficult (e.g., higher whole-body coordination requirements) are related to greater executive functions compared to easy motor tasks [75]. Interestingly, executive functions appear to become more involved in performing novel motor competence tasks and decrease when motor competence tasks are learned [15]. Future exploration of the influence of the cognitive demands of performing motor tasks (e.g., comparisons of a novice and someone already proficient in the movement) may enhance our understanding of the relationship between motor competence and executive functions in children and adolescents.

## Conclusions

The findings from our review suggest a small positive association between motor competence and executive functions in children and adolescents. Specifically, all types of motor competence (e.g., locomotor, object control and stability skills) were significantly associated with each domain of executive functioning (e.g., inhibition, working memory, cognitive flexibility). The small number of experimental studies included in this review support the assertion that interventions targeting children’s motor competence may be a promising strategy to improve their executive functions; however, more research is needed to confirm these findings. Future studies should explore the underlying mechanisms linking motor competence and executive functions as their comprehension may be used to optimise future intervention design and delivery.

### Supplementary Information

Below is the link to the electronic supplementary material.

Supplementary file1 (DOCX 14 KB)

Supplementary file2 (DOCX 43 KB)

Supplementary file3 (DOCX 16 KB)

Supplementary file4 (R 3 KB)

Supplementary file5 (DOCX 442 KB)

## Declarations

### Availability of Data and Material

All data relevant to the study are included in the article or uploaded as supplementary information.

### Code Availability

*R* script used for the meta-analysis is available in the online supplementary material.
