# Supplementary material for: Associations Between Motor Competence and Executive Functions in Children and Adolescents: A Systematic Review and Meta-analysis
Source: Sports Med. 2024 May 20;54(8):2141–56. doi: 10.1007/s40279-024-02040-1 (PMC11329584; doi:10.1007/s40279-024-02040-1)
Supplement: Supplementary file 1 — Supplementary file1 (DOCX 14 KB) [file 40279_2024_2040_MOESM1_ESM.docx]

Systematic Review and Meta-Analysis of the Associations between Motor Competence and Executive Functions in Children and Adolescents

Ran Bao^1, 2, 3^, Levi Wade^1, 2, 3^, Angus A. Leahy^1, 2, 3^, Katherine B. Owen^4^, Charles H. Hillman^5^, Timo Jaakkola^6^, David R. Lubans^1, 2, 3, 6,^ *

^1^Centre for Active Living and Learning, University of Newcastle, Callaghan, New South Wales, Australia

^2^ College of Human and Social Futures, School of Education, University of Newcastle, Callaghan, New South Wales, Australia

^3^ Active Living Research Program, Hunter Medical Research Institute, New Lambton Heights, New South Wales, Australia

^4^ SPRINTER, Prevention Research Collaboration, Level 6, Charles Perkins Centre, School of Public Health, Faculty of Medicine and Health, The University of Sydney, Sydney, New South Wales, Australia.

^5^ Department of Psychology, Department of Physical Therapy, Movement, & Rehabilitation Sciences, Northeastern University, Boston, Massachusetts, USA

^6^ Faculty of Sport and Health Sciences, University of Jyväskylä, Jyvaskyla, Finland

Corresponding author *:

David Revalds Lubans

[david.lubans@newcastle.edu.au](mailto:david.lubans@newcastle.edu.au)

Table S1. Search terms used in this systematic review and meta-analysis

| Keywords | Search terms |
| --- | --- |
| Motor competence | *motor competenc* OR motor skills OR motor performance OR motor proficiency OR fundamental movement skills OR fundamental motor skills OR motor coordination OR motor function OR motor development OR motor abilities OR motor control OR motor examination OR motor milestones OR motor behavio* OR gross motor skills OR actual competence OR object control OR object manipulation OR locomotor skill OR stability OR balance OR postural control OR stability skills OR movement assessment battery* |
| Executive functions | *cognitive skills OR cognitive performance OR cognitive function OR cognitive control OR cognitive processes OR cognition OR executive function OR executive control OR inhibition OR selective attention OR interference control OR working memory OR cognitive flexibility OR mental flexibility OR task switching* |
| Population | *child* OR adolescen* OR kindergarten OR teen* OR youth OR young people OR young person OR student** |
