# Supplementary material for: Associations Between Motor Competence and Executive Functions in Children and Adolescents: A Systematic Review and Meta-analysis
Source: Sports Med. 2024 May 20;54(8):2141–56. doi: 10.1007/s40279-024-02040-1 (PMC11329584; doi:10.1007/s40279-024-02040-1)
Supplement: Supplementary file 2 — Supplementary file2 (DOCX 43 KB) [file 40279_2024_2040_MOESM2_ESM.docx]

Systematic Review and Meta-Analysis of the Associations between Motor Competence and Executive Functions in Children and Adolescents

Ran Bao^1, 2, 3^, Levi Wade^1, 2, 3^, Angus A. Leahy^1, 2, 3^, Katherine B. Owen^4^, Charles H. Hillman^5^, Timo Jaakkola^6^, David R. Lubans^1, 2, 3, 6,^ *

^1^Centre for Active Living and Learning, University of Newcastle, Callaghan, New South Wales, Australia

^2^ College of Human and Social Futures, School of Education, University of Newcastle, Callaghan, New South Wales, Australia

^3^ Active Living Research Program, Hunter Medical Research Institute, New Lambton Heights, New South Wales, Australia

^4^ SPRINTER, Prevention Research Collaboration, Level 6, Charles Perkins Centre, School of Public Health, Faculty of Medicine and Health, The University of Sydney, Sydney, New South Wales, Australia.

^5^ Department of Psychology, Department of Physical Therapy, Movement, & Rehabilitation Sciences, Northeastern University, Boston, Massachusetts, USA

^6^ Faculty of Sport and Health Sciences, University of Jyväskylä, Jyvaskyla, Finland

Corresponding author *:

David Revalds Lubans

david.lubans@newcastle.edu.au

Table S2. The characteristics of the included studies

| **Study** | **Sample** | **Design** | **MC measure** | **EFs measure** | **Results** |
| --- | --- | --- | --- | --- | --- |
| Aadland 2017 | N = 697  357 girls and 340 boys  Mean age = 10.2±0.3 years  Norway | Cross-sectional | Object control skills (MABC-2):  (1) catching with one hand  (2) throwing at a wall target | (1) Stroop Colour and Word Test: inhibition  (2) the trail making test: cognitive flexibility  (3) Digit Span test (Backward) and: Working memory | For boys and girls, motor competence was positively associated with inhibition, working memory, cognitive flexibility, and overall executive functions. |
| Albuquerque 2021 | N = 152  67 boys and 85 girls  Age range = 6-11 years  Mean age (6-8) = 7.1 ± 0.7 years  Mean age (9-11) = 9.8 ± 0.8 years  Brazil | Cross-sectional | Gross motor skills  (1) locomotor skills (KTK):  walking backward, shifting platforms, jumping on one leg over an obstacle, and jumping laterally.  (2) TGMD-2:  a. locomotor (run, jump, gallop, hop, slide, leap)  b. object control (strike, bounce, throw, kick, catch, roll a ball). | (1) The computerized version of the Flanker/Reverse Flanker: executive functions  (2) Tower of London (TOL): general executive functions | Motor competence was positively associated with executive functions in children. |
| Biino 2021 | N = 36  17 boys and 19 girls  Mean age = 60.6±7.4 months  Italy | Non-randomised trial  (12 weeks) | Peabody Developmental Motor Scale-Second Edition: reflexes in children up to 11 months (eight items), stationary performance (30 items), locomotion (89 items) and object manipulation skills (24 items) | (1) the Word List Recall test: working memory  (2) the Trail Making Test for young children (TRAILS-P) tests: inhibition, cognitive flexibility | Motor competence was positively associated with working memory, inhibition, and cognitive flexibility. |
| Capio 2022 | N = 107  44 boys and 63 girls  Mean age = 5.84 ± 0.67 years  Hong Kong, China | Cross-sectional | TGMD-2:  (1) locomotor (run, jump, gallop, hop, slide, leap)  (2) object control (strike, bounce, throw, kick, catch, roll a ball) | (1) the backward digit recall test: verbal working memory  (2) the backward Corsi block tapping test: visuospatial working memory | Object control skills were significantly associated with greater verbal working memory |
| De Bruijn 2023 | N = 891  440 boys and 451 girls  Mean age = 9.17 ± 0.66 years  Netherlands | Cross-sectional | KTK:  (1) locomotor skills (jumping laterally and shifting platforms)  (2) stability skills: balancing backwards (balancing skill)  BOT-2:  (3) object control skills: ball skills | (1) the Stop Signal Task (SST): response inhibition  (2) the Digit span task (digit span backward): verbal working memory | Motor competence was positively associated with working memory and response inhibition |
| Eriksen 2023 | N = 85  43 boys and 42 girls  Age range = 6-7 years  Mean age = 6.77 ± 3.15 years  Norway | Cross-sectional | Stability skills: (the test of Motor Competence):  (1) dynamic balance (walking/running in slopes (W/R) and heel to toe walking (HTW)) | The computer-based test Spanboard: visuospatial working memory | No significant association was found between motor competence and working memory |
| FERNANDEZ-SANCHEZ 2022 | N = 451  217 boys and 234 girls  Age range = 8-10 years  Mean age = 9.95 ± 0.59 years  Spain | Cross-sectional | MABC-2:  (1) object control skills: aiming and catching  (2) stability skills: static and dynamic balance | (1) the Flanker task: inhibitory control  (2) the Dimensional Chang Card Sort test: cognitive flexibility  (3) the List Sorting Working Memory test: working memory | Gross motor competence was significantly associated with working memory, inhibition, and cognitive flexibility. |
| Geertsen 2016 | N = 423  214 boys and 209 girls  Mean age = 9.29±0.35 years  Age range = 8-10 years  Denmark | Cross-sectional | Stability skills (balance): coordination wall | The spatial working memory test: spatial working memory | Motor competence (motor-skill wall) was positively associated with working memory |
| Jansen 2019 | N = 185 (90 Germany and 95 Oman)  Germany: 48 boys and 42 girls  Mean age = 8.6±0.61 yeas  Oman: 48 boys and 47 girls  Mean age = 7.95±0.55 years  German and Oman | Cross-sectional | Object control skills: (the German General Motor Test):  (1) Target throw (precision task) | The CorsiBlock-TappingTest (forward and backwards) and the Digit-SpanTest (forward and backwards): working memory | Motor competence (target throw) was positively associated with working memory (Digit-Span forward) |
| Klotzbier 2022 | N = 12 CA children (6 boys and 6 girls) and 12 MA (6 boys and 6 girls)  Mean age (CA) = 10.5±10.07 years  Mean age (MA) = 5.98± 1.21 years  Age range = 4-11 years  Germany | Cross-sectional | MABC-2:  (1) object control skills: aiming and catching  (2) stability skills: balance | The Trail-Making Test-B: executive function | The positive association between motor competence (aiming and catching, balance) and inhibition. |
| Lehmann 2014 | N = 65  32 boys and 33 girls  Mean age (girls) = 5.13±0.89 years  Mean age (boys) = 4.86±1.04 years  Age range = 3-6 years  Germany | Cross-sectional | MABC-2:  (1) object control skills: ball skills (throw and catch a sandbag)  (2) stability skills: static and dynamic balance (one leg stand, walking with heels up, mat bouncing) | Digit span forward and backward, Corsi block tapping test forward and backward: working memory | Motor competence (balance) was positively associated with working memory. |
| Liu 2022 | N = 364  Girls (46.1%)  Age range = 9-10 years  Mean age = 9.55 ± 0.92 years  China | Cross-sectional | KTK:  (1) locomotor skills: jumping sideways; moving sideways  (2) stability skills: balance (walking backward) | (1) the flanker task: inhibition  (2) 1-back task: working memory  (3) the more-odd-shifting task: cognitive flexibility | A week correlation was found between motor competence and inhibition and working memory reaction time. |
| Livesey 2006 | N = 36  15 boys and 21 girls  Mean age = 75 months  Age range = 5-6 years  Australia | Cross-sectional | MABC:  (1) object control skills: ball skills  (2) stability skills: balance | Stroop and stop-signal task: inhibition  Day night Stroop task: interference control | Motor competence (ball skills) was positively associated with inhibition. |
| Ludyga 2018 | N = 82  48 boys and 34 girls  Mean age = 11.2±1.2 years  Age range = 10-15 years  Switzerland | Cross-sectional | MOBAK-5:  (1) object control skills (bouncing, dribbling, throwing, and catching)  (2) locomotor skills (rolling, jumping, and running)  (3) stability skills: balancing | A modified Sternberg task: working memory | Motor competence (locomotor and object control skills) was positively associated with working memory. |
| Ludyga 2019 | N = 89  45 boys and 44 girls  Mean age = 11.3±0.5 years  Age range = 10-12 years  Switzerland | Cross-sectional | MOBAK-5:  (1) object control skills (throwing, catching, bouncing, dribbling)  (2) locomotor skills (rolling, rope skipping, running)  (3) stability skills: balancing | (1) the flanker task: inhibition  (2) the mixed flanker test: cognitive flexibility,  (3) 2-Back task: working memory | Object control skills was positively associated with inhibition and cognitive flexibility, locomotor skills was positively related to working memory. |
| Ludyga 2020 | N = 52  27 boys and 25 girls  Mean age = 10.27±0.45 years  Age range = 10-12 years  Germany | Longitudinal | The MOBAK-5:  (1) locomotor skills (rolling, jumping, running)  (2) object control (bouncing, dribbling, throwing, and catching  (3) stability skills: balancing | The Sternberg task (modified version): visual working memory | Motor competence (FMS_total_) was positively associated with working memory. |
| Luz 2015 | N = 96  53 boys and 43 girls  Mean age = 9.99±0.34 years  Age range = 9-11 years  Portugal | Cross-sectional | KTK:  (1) locomotor skills: hopping, jumping, shifting  (2) stability skills: balance | Planning scale from the Cognitive Assessment System: matching numbers, planned codes, planned connections, planning scale | Motor competence was positively associated with executive functions. |
| Maurer 2019 | N = 124  57 boys and 67 girls  Mean age = 71±5.8 months  Age range = 5-6 years  Switzerland | Cross-sectional | Locomotor skills (KTK): jumping sideways, moving sideways  Stability skills (MABC-2): balance (one-leg-stand) | (1) a modified version of the Flanker task: inhibition,  (2) an adapted computer-based pictorial updating task: working memory (updating)  (3) an adapted Advanced Dimensional Change Card Sort task: cognitive flexibility | Motor competence (jumping sideways) was positively associated with inhibition and working memory (updating). |
| Mazzoccante 2020^a^ | N = 271  45.75% boys  Mean age = 7.53± 1.52 years  Age range = 6-10 years  Brazil | Cross-sectional | KTK:  (1) locomotor skills: hopping for height, jumping sideways over a slat, moving sideways on boards  (2) stability skills: balance (walking backward along balance beams of different widths) | Visual search of pencil and paper tests (trail making test): cognitive flexibility | Motor competence was positively associated with overall executive functions. |
| Mazzoccante 2020^b^ | N = 68  (1) sports (N = 34, 22 boys, 12 girls)  (2) Non-sports (N = 34, 8 boys, 26 girls)  Mean age (sports) = 6.94±0.23 years  Mean age (non-sports) = 6.64±0.48 years  Age range = 6-7 years  Brazil | Cross-sectional | KTK:  (1) locomotor skills: monopodial jump, transfer on platforms and lateral jumps  (2) stability skills: balance beam | Cognitive flexibility: trail making test A and B | Motor competence was positively associated with executive functions. |
| Meijer 2021 | N = 92  46 boys and 46 girls  Mean age = 9.12±0.62 years  Age range = 8-10.7 years  Netherlands | Cross-sectional | (1) locomotor skills (KTK): jumping sideways, moving sideways  (2) object control skills (BOT-2): ball skills  (3) stability skills (KTK): backward balancing | (1) Digit span and grid task: working memory  (2) stop signal task: inhibition (attention efficiency) | Motor competence was positively associated with visuospatial working memory, inhibition, verbal working memory. |
| Meijer 2022 | N = 90  45 boys and 45 girls  Mean age = 9.13 ± 0.62  Age range = 8-11 years  Netherland | Cross-sectional | (1) locomotor skills (KTK): jumping sideways, moving sideways  (2) object control skills (BOT-2): ball skills  (3) stability skills (KTK): backward balancing | (1) the stop signal task: inhibition  (2) the digit span: verbal working memory | Gross motor competence was significantly associated with motor inhibition. |
| Michel 2019 | N = 173  57% boys  Mean age = 65±7 months  Age range = 51-84 months  Germany | Cross-sectional | MABC-2:  (1) object control skills: the ball skills scale (catching bean bag, throwing bean back onto mat)  (2) stability skills: the balance scale (one-leg balance, walking heels raised, jumping on mats) | (1) Flanker task: inhibition  (2) Go/NoGo task: inhibition  (3) Colour span backwards: working memory  (4) Corsi-blocks backwards: Visual-spatial dynamic working memory | Motor competence (balance) was positively associated with inhibition, visual-spatial dynamic working memory. |
| Mulvey 2018 | N = 107 (50 intervention, 57 control)  54.2% girls  Mean age = 5.14±0.81 years  Age range = 3-6 years  United States | RCT | TGMD-2:  (1) locomotor skills (running, galloping, sliding, hopping, jumping, sliding)  (2) object control skills (dribbling, kicking, striking, catching, throwing, rolling) | The HTKS task: executive functions | Motor competence intervention (SKIP group) effectively improve children’s executive functions. |
| Niederer 2011 | N = 245  49.4% girls  Mean age = 5.2±0.6 years  German and Switzerland | Cross-sectional and Longitudinal (9 months) | Stability skills (balance):  (1) balancing forward bare-foot on a 3 m long and 3 cm wide  (2) balance beam: dynamic balance | Spatial working memory test from the Intelligence and Development Scales (IDS): spatial working memory | For longitudinal associaiton, balance (baseline) was positively related to working memory (9 months later). |
| Oberer 2017 | N = 156  51.0% girls  Mean age = 6 years and 5 months (SD = 4 months)  Age range = 68-87 months  Switzerland | Cross-sectional | (1) locomotor skills (KTK): jumping sideways, moving sideways  (2) stability skills (MABC-2): balance (one-leg-stand) | (1) an adapted version of the flanker task: inhibition  (2) the flanker mixed test: cognitive flexibility  (2) the backwards colour recall task: working memory (updating) | Motor competence (jumping sideways, moving sideways) was positively associated with inhibition, cognitive flexibility and working memory. Balance was positively related to inhibition and working memory. |
| O’Hagan 2022 | N = 60  Girls (51.7%)  Mean age = 9.9 ± 1.28 years  Age range = 7-12 years  Ireland | Cross-sectional | (1) locomotor (TGMD-3) skills: run, skip, gallop, slide, hop, and horizontal jump  (2) object control skills (TGMD-3): catch, overhand throw, underhand roll, kick, two-handed strike, one-handed strike, and stationary dribble  (3) stability skills (BOT-2): balance | The Cambridge Neuropsychological Test Automated Battery: spatial working memory (SWM) | There was no significant association between gross motor competence and working memory |
| Policastro 2018 | N = 75  53 boys and 22 girls  Mean age = 9.36±0.98 years  Age range = 7.23-10.99 years  Italy | Cross-sectional | MABC-2:  (1) object control skills: aiming and catching  (2) stability skills: balance | NEPSY-II: Inhibition and Switching  Corsi's Test-Sequential Spatial Task: visuospatial short-term working memory | Motor competence was positively associated with inhibition and working memory. |
| Rigoli 2012^a^ | N = 93  55 boys and 38 girls  Mean age = 14 years 2 months (SD = 1 years 1 month)  Age range = 12-14 years  Australia | Cross-sectional | MABC-2:  (1) object control skills: aiming and catching  (2) stability skills: balance | (1) N-back task: visuospatial working memory  (2) inhibition: Inhibition task from NEPSY-II  (3) cognitive flexibility: Switching task from NEPSY-II | Motor competence was positively associated with inhibition, cognitive flexibility and working memory. |
| Rigoli 2012^b^ | N = 93  55 boys and 38 girls  Mean age = 14.2±1.1 years  Age range = 12-16 years Australia | Cross-sectional | MABC-2:  (1) object control skills: aiming and catching  (2) stability skills: balance | N-back task: working memory | Motor competence (aiming and catching) |
| Rigoli 2013 | N = 161  73 boys and 88 girls  Age range = 5-11 years  Mean age = 8.56 ± 1.46 years  Australia | Longitudinal  (18 months) | Locomotor skills (McCarron Assessment of Neuromuscular Development)  (1) Hand Strength (right and left hand)  (2) Finger-Nose-Finger (eyes open and closed)  (3) Jumping  (4) Heel-Toe-Walk (forward and backward)  (5) Standing on One Foot (eyes open and closed on each leg) | One-Back task- CogState Brief Battery (CogState Ltd., Melbourne, Australia):  Visual working memory | There was an insignificant association between motor competence (gross motor skills) and working memory (one-back task). |
| Roebers 2009 | N = 112  52.7% girls  Mean age = 7 years and 6 months  Age range = 79-106 months  Switzerland | Cross-sectional | Locomotor skills: jumping, moving sideways | (1) Backward Colour recall task  (2) the Flanker task  (3) the Simon task  (4) the Cognitive Flexibility task | Jumping was positively associated with inhibition (Simon RT) and cognitive flexibility (RT), moving sideways positively related to cognitive flexibility (RT). |
| Rudd 2021 | N = 31  46% girls  Mean age = 6.6±0.5 years  Age range = 6-7 years  Australia | Longitudinal and RCT | The Canadian Agility and Movement Skill Assessment:  (1) locomotor skills: two-feet jumping inside hoops, sliding sideways, skipping, one-foot jumping inside hoops  (2) object control skills: catching and throwing, kicking a ball | (1) list sorting working memory: working memory  (2) dimensional change card sort: cognitive flexibility  (3) Flanker: inhibitory control | Motor competence was positively associated with inhibition and working memory. |
| Scharfen 2019 | N = 15  Mean age = 12.72±0.45 years  Age range = 11-13 years  Germany | Cross-sectional | Object control skills:  (1) dribbling (the Dribbling test)  (2) ball control (the Ball Control test)  (3) ball juggling (the Ball Juggling test) | Working memory span test (digit span test): working memory | Motor competence was positively associated with working memory. |
| Schmidt 2017 | N = 236  52.5% girls  Mean age = 11.3±0.6 years  Age range = 10-12 years  Switzerland | Longitudinal (10 weeks) | Locomotor skills (KTK): jumping sideways) | (1) a non-spatial n-back task: working memory (updating)  (2) a child-adapted Eriksen flanker task: inhibition  (3) the flanker task ("Mixed block"): cognitive flexibility | Motor competence (jumping sideways) was positively associated with inhibition, cognitive flexibility and working memory. |
| Spanou 2022 | N = 115  42.61% boys  Mean age = 10.3 ±1.2 years  Age range = 8-12 years  Greece | Cross-sectional | BOT-2SF:  (1) locomotor skills: walking forward on a line;; one-legged stationary hop  (2) object control skills: dropping and catching a ball; dribbling a ball standing (3) stability skills: on one leg on a balance beam-eyes open | (1) “How many—What number” testing task (mixed block): cognitive flexibility  (2) the digits span (backwards): working memory | Motor competence was significantly associated with cognitive flexibility and working memory |
| Stein 2017 | N = 101  Mean age = 60-85 months  Intervention: N = 48 (24 girls)  Mean age = 72.2 ±5.2 months  Control: N = 53 (28 girls)  Mean age = 72.3 ±6.9 months  Germany | RCT | M-ABC 2:  (1) object control skills: ball skills  (2) stability skills: balance | (1) the Simon-says task: motor inhibition  (2) the computer-based Hearts-and-Flowers task: inhibition and cognitive flexibility | Ball skills were positively associated with cognitive flexibility (accuracy) and cognitive inhibition (reaction time), dynamic balance was positively related to motor inhibition (accuracy) and cognitive flexibility (accuracy). |
| Stuhr 2020 | N = 41  18 boys and 23 girls  Mean age = 71.9±3.9 months  Age range = 5-6 years  Germany | Cross-sectional | Stability skills (SEBT): balance | (1) List-Sorting test: working memory  (2) Hearts and Flowers task: response inhibition  (3) Flanker task  (4) Wisconsin Card Sorting task: cognitive flexibility | An insignificant association between balance and executive functions was found in younger children. |
| SyvÃ¤oja 2021 | N = 309  59% girls  Mean age = 14.0 years  Age range = 12-17  Finland | Cross-sectional | (1) locomotor skills (the 5-leaps test): jumping  (2) object control skills (throwing-catching combination test): throwing, catching | (1) A modified version of the Eriksen flanker task: inhibition  (2) Spatial Working Memory test (SWM): visuospatial working memory  (3) Digit Span test: auditory-verbal working memory | For overall sample, motor competence was positively associated with inhibition. For girls, motor competence was significantly related to visuospatial working memory. |
| Trecroci 2021 | N = 43  43 girls  Mean age = 11.2±0.8 years  Italy | Cross-sectional | Stability skills (the balance error scoring system test): balance | A modified version of the Flanker task with arrows: executive control | Motor competence (balance) was positively associated with inhibition. |
| vanderFels 2019 | N = 732  50.0% girls  Mean age = 9.16±0.64 years  Age range = 8-10 years  Netherland | Cross-sectional | (1) locomotor skills (KTK): jumping sideways, moving sideways  (2) object control skills (BOT-2): ball skills  (3) stability skills: backwards balancing | (1) Visuospatial Working Memory task: verbal and visuospatial working memory  (2) Verbal working memory: Digit Span Backward of the Digit Span task of the WISC-III  (3) Stop Signal task: response inhibition  (3) Flanker task: interference control | Motor competence was positively associated with verbal working memory, visuospatial working memory, response inhibition, interference control. |
| vanderFels 2020 | N = 80  51.3% girls  Mean age = 9.14±0.63 years  Age range = 8-10 years  Netherland | Cross-sectional | (1) locomotor skills (KTK): jumping sideways, moving sideways  (2) object control skills (BOT-2): ball skills  (3) stability skills: backwards balancing | An adapted version of a spatial span task: visuospatial working memory | Motor competence was positively associated with working memory. |
| Vazou 2020 | N = 34  21 boys and 13 girls  Mean age = 7.69±1.52 years  Age range = 6-11 years  United States | Non-randomised trial (7 weeks) | MABC-2:  (1) locomotor skills: walking on a line using tandem foot placement without stepping off the line for a maximum of 15 steps (2 trials), hopping in a controlled manner up to 5 continuous hops on a series of preplaced squares (2 trials)  (2) stability skills: balancing on one foot on a balance board for up to 30 s (one trial on each leg) | (1) the standard Flanker test: inhibition  (2) the mixed flanker test: cognitive flexibility | Motor competence (balance) was positively associated with cognitive flexibility. |
| Wassenberg 2005 | N = 378  213 boys and 165 girls  Mean age = 6.18±0.45 years  Netherland | Cross-sectional | The Maastricht Motor Test:  (1) object control skills: ball skills  (2) stability skills: dynamic balance, static balance | The Number Recall test and the Word Order test of the K-ABC: auditory working memory | Positive association between motor competence and working memory. |

Note: STG = a single cognitive task group, DTG = a dual-task training group; FMS = fundamental movement skills, OC = object control skills, LM = locomotor skills; CA = typical children with the same chronological age, MA = typical children with the same mental age; MABC-2 = Movement assessment battery for children 2; KTK = Körperkoordinationstest Für Kinder; BOT-2 = Bruininks-Oseretsky Test of Motor Proficiency; SEBT = Star Excursion Balance test; MOBAK-5 = Motorische Basiskompetenzen-fifth test battery; TGMD-2 = The Test of Gross Motor Development–Second Edition; T1 = baseline; T2 = follow-up; Rigoli 2012^a^ = An examination of the relationship between motor coordination and executive functions in adolescents; Rigoli 2012^b^ = Motor coordination, working memory, and academic achievement in a normative adolescent sample: Testing a mediation model; RCT = randomised control trial; Mazzoccante 2020^a^ = Attention and executive function are predicted by anthropometric indicators, strength, motor performance, and aerobic fitness in children aged 6-10 years; Mazzoccante 2020^b^ = The influence of sports practice in children aged 6-7 years on physical fitness, motor coordination and executive functions.
