# Supplementary material for: Associations Between Motor Competence and Executive Functions in Children and Adolescents: A Systematic Review and Meta-analysis
Source: Sports Med. 2024 May 20;54(8):2141–56. doi: 10.1007/s40279-024-02040-1 (PMC11329584; doi:10.1007/s40279-024-02040-1)
Supplement: Supplementary file 3 — Supplementary file3 (DOCX 16 KB) [file 40279_2024_2040_MOESM3_ESM.docx]

Systematic Review and Meta-Analysis of the Associations between Motor Competence and Executive Functions in Children and Adolescents

Ran Bao^1, 2, 3^, Levi Wade^1, 2, 3^, Angus A. Leahy^1, 2, 3^, Katherine B. Owen^4^, Charles H. Hillman^5^, Timo Jaakkola^6^, David R. Lubans^1, 2, 3, 6,^ *

^1^Centre for Active Living and Learning, University of Newcastle, Callaghan, New South Wales, Australia

^2^ College of Human and Social Futures, School of Education, University of Newcastle, Callaghan, New South Wales, Australia

^3^ Active Living Research Program, Hunter Medical Research Institute, New Lambton Heights, New South Wales, Australia

^4^ SPRINTER, Prevention Research Collaboration, Level 6, Charles Perkins Centre, School of Public Health, Faculty of Medicine and Health, The University of Sydney, Sydney, New South Wales, Australia.

^5^ Department of Psychology, Department of Physical Therapy, Movement, & Rehabilitation Sciences, Northeastern University, Boston, Massachusetts, USA

^6^ Faculty of Sport and Health Sciences, University of Jyväskylä, Jyvaskyla, Finland

Corresponding author *:

David Revalds Lubans

david.lubans@newcastle.edu.au

| Table S3. List of the measures used by the studies included in this review, organised by outcomes. Note that many cognitive tests span multiple aspects of executive functions, as indicated below. | |
| --- | --- |
| **Outcome** | **Measure** |
| *Executive function* | |
| Cognitive flexibility | The Trail-Making Test (A and B)  Trail Making Test for young children (TRAILS-P) test  The Dimensional change card sort (DCSS) test  The Additional block (“mixed” block) included in the flanker task  The Cognitive Flexibility task  The Mixed Flanker test  The Wisconsin Card Sorting test (WCST)  The Switching task from NEPSY-II  The more-odd-shifting task |
| Inhibition | The HTKS task  The Flanker/Reverse/Mixed/ Eriksen/modified Flanker test  The Child-adapted Eriksen flanker task  The Visual Discrimination task  The Simon task  The Go/NoGo task  The Stop Signal task  The Day and night stroop task  The Simon-says task (motor inhibition)  The Stroop Colour and Word Test  The Hearts and Flowers Test  The Naming and Inhibition task from NEPSY-II |
| Working memory | The Rapid Visual Processing  The One-Back task-CogState Brief Battery  The Word List Recall test  The List sorting working memory test  The Modified Sternberg task  The N-Back tasks  The Digit span (forward and backward) test  The Corsi block tapping test forward and backward  The Corsi’s Test—Sequential Spatial Task  The Non-spatial n-back task (Updating)  The Corsi Block Test (visual-spatial updating)  The Spatial Working Memory test  The Backward Colour recall task  The Colour span backwards  The Digit Span task (backward) of the WISC  The Number Recall test and the Word Order test of the K-ABC (auditory working memory)  The Visuospatial Memory task  The Working memory span test  An adapted version of a spatial span task  The Toolbox List Sorting Working Memory Test  The computer-based pictorial updating task (updating)  The HAWIK-IV (Working memory scale from Intelligence and Development Scales (IDS))  The spatial working memory test from the Cambridge Neuropsychological Test Automated Battery |
| General executive function | The computer-based “Hearts-and-Flowers task”  The Tower of London task  The NEPSY-II  The Planning scale from the CAS |
