# Supplementary material for: Associations Between Motor Competence and Executive Functions in Children and Adolescents: A Systematic Review and Meta-analysis
Source: Sports Med. 2024 May 20;54(8):2141–56. doi: 10.1007/s40279-024-02040-1 (PMC11329584; doi:10.1007/s40279-024-02040-1)

Systematic Review and Meta-Analysis of the Associations between Motor Competence and Executive Functions in Children and Adolescents

Ran Bao^1, 2, 3^, Levi Wade^1, 2, 3^, Angus A. Leahy^1, 2, 3^, Katherine B. Owen^4^, Charles H. Hillman^5^, Timo Jaakkola^6^, David R. Lubans^1, 2, 3, 6,^ *

^1^Centre for Active Living and Learning, University of Newcastle, Callaghan, New South Wales, Australia

^2^ College of Human and Social Futures, School of Education, University of Newcastle, Callaghan, New South Wales, Australia

^3^ Active Living Research Program, Hunter Medical Research Institute, New Lambton Heights, New South Wales, Australia

^4^ SPRINTER, Prevention Research Collaboration, Level 6, Charles Perkins Centre, School of Public Health, Faculty of Medicine and Health, The University of Sydney, Sydney, New South Wales, Australia.

^5^ Department of Psychology, Department of Physical Therapy, Movement, & Rehabilitation Sciences, Northeastern University, Boston, Massachusetts, USA

^6^ Faculty of Sport and Health Sciences, University of Jyväskylä, Jyvaskyla, Finland

Corresponding author *:

David Revalds Lubans

david.lubans@newcastle.edu.au

**Table S4** The risk of bias assessment of the included studies (cross-sectional studies)

| First author | Q1 | Q2 | Q3 | Q4 | Q5 | Q6 | Q7 | Q8 | Yes (%) | RoB |
| --- | --- | --- | --- | --- | --- | --- | --- | --- | --- | --- |
| (1) Aadland 2017 | ✓ | ✓ | ✓ | NA | ✓ | ✓ | ✓ | ✓ | 100.0% | Low |
| (2) Albuquerque 2021 | ✓ | ✓ | ✓ | NA | ✓ | ✓ | ✓ | ✓ | 100.0% | Low |
| (3) Geertsen 2016 | x | ✓ | ? | NA | ✓ | ✓ | ? | ✓ | 57.1% | Moderate |
| (4) Jansen 2019 | ✓ | ✓ | ? | NA | x | x | ✓ | ✓ | 57.1% | Moderate |
| (5) Klotzbier 2022 | ✓ | ✓ | ✓ | NA | ✓ | ✓ | ✓ | ✓ | 100.0% | Low |
| (6) Lehmann 2014 | x | ✓ | ✓ | NA | ✓ | ✓ | ✓ | ✓ | 85.7% | Low |
| (7) Livesey 2006 | x | ✓ | ✓ | NA | ✓ | ✓ | ? | ✓ | 71.4% | Low |
| (8) Ludyga 2018 | ✓ | ✓ | ✓ | NA | ✓ | ✓ | ✓ | ✓ | 100.0% | Low |
| (9) Ludyga 2019 | ✓ | ✓ | ✓ | NA | ✓ | ✓ | ✓ | ✓ | 100.0% | Low |
| (10) Luz 2015 | ✓ | ✓ | ✓ | NA | ✓ | ✓ | ✓ | ✓ | 100.0% | Low |
| (11) Maurer 2019 | ✓ | ✓ | ✓ | NA | ✓ | ✓ | ✓ | ✓ | 100.0% | Low |
| (12) Mazzoccante 2020***^a^*** | ✓ | ✓ | ✓ | NA | x | x | ✓ | ✓ | 71.4% | Low |
| (13) Mazzoccante 2020***^b^*** | ✓ | ✓ | ✓ | NA | x | x | ✓ | ✓ | 71.4% | Low |
| (14) Meijer 2021 | ✓ | ✓ | ✓ | NA | ✓ | ✓ | ✓ | ✓ | 100.0% | Low |
| (15) Oberer 2017 | x | ✓ | ✓ | NA | ✓ | ✓ | ✓ | ✓ | 85.7% | Low |
| (16) Policastro 2018 | ✓ | ✓ | ✓ | NA | ✓ | ✓ | ✓ | ✓ | 100.0% | Low |
| (17) Rigoli 2012^a^ | ✓ | ✓ | ✓ | NA | ✓ | ✓ | ✓ | ✓ | 100.0% | Low |
| (18) Rigoli 2012^b^ | ✓ | ✓ | ✓ | NA | ✓ | ✓ | ✓ | ✓ | 100.0% | Low |
| (19) Roebers 2009 | ✓ | ✓ | ? | NA | ✓ | ✓ | ? | ✓ | 71.4% | Low |
| (20) Scharfen 2019 | ✓ | ✓ | ✓ | NA | x | x | ? | ✓ | 57.1% | Moderate |
| (21) Stuhr 2020 | ✓ | ✓ | ✓ | NA | ✓ | ✓ | ✓ | ✓ | 100.0% | Low |
| (22) SyvÃ¤oja 2021 | ✓ | ✓ | ✓ | NA | ✓ | ✓ | ✓ | ✓ | 100.0% | Low |
| (23) Trecroci 2021 | x | ✓ | ✓ | NA | x | x | ✓ | ✓ | 57.1% | Moderate |
| (24) VanderFels 2019 | ✓ | ✓ | ✓ | NA | ✓ | ✓ | ✓ | ✓ | 100.0% | Low |
| (25) VanderFels 2020 | ✓ | ✓ | ✓ | NA | x | x | ? | ✓ | 57.1% | Moderate |
| (26) Wassenberg 2005 | ✓ | ✓ | ✓ | NA | ✓ | ✓ | ✓ | ✓ | 100.0% | Low |
| (27) Capio 2022 | ✓ | ✓ | ✓ | NA | x | x | ✓ | ✓ | 71.4% | Low |
| (28) De Bruijn 2023 | x | ✓ | ✓ | NA | x | x | ✓ | ✓ | 57.1% | Moderate |
| (29) Eriksen 2023 | ✓ | ✓ | ✓ | NA | x | x | ? | ✓ | 57.1% | Moderate |
| (30) FERNANDEZ-SANCHEZ 2022 | x | ✓ | ✓ | NA | x | x | ✓ | ✓ | 57.1% | Moderate |
| (31) Liu 2022 | ✓ | ✓ | ✓ | NA | x | x | ✓ | ✓ | 71.4% | Low |
| (32) Meijer 2022 | ✓ | ✓ | ✓ | NA | ✓ | ✓ | ✓ | ✓ | 100.0% | Low |
| (33) O’Hagan 2022 | ✓ | ✓ | ✓ | NA | x | x | ? | ✓ | 57.1% | Moderate |
| (34) Spanou 2022 | ✓ | ✓ | ✓ | NA | x | x | ? | ✓ | 57.1% | Moderate |

Note: Mazzoccante 2020***^a^*** = Attention and executive function are predicted by anthropometric indicators, strength, motor performance, and aerobic fitness in children aged 6-10 years; Mazzoccante 2020***^b^*** =The influence of sports practice in children aged 6 to 7 years on physical fitness, motor coordination and executive functions; Rigoli 2012^a^ = An examination of the relationship between motor coordination and executive functions in adolescents; Rigoli 2012^b^ = Motor coordination, working memory, and academic achievement in a normative adolescent sample: Testing a mediation model; x = “no”, ✓= “yes”, ? = “unclear”, NA = “not applicable”.

**Table S5** The risk of bias assessment of the included studies (longitudinal studies)

| First author | Q1 | Q2 | Q3 | Q4 | Q5 | Q6 | Q7 | Q8 | Q9 | Q10 | Q11 | Yes (%) | RoB |
| --- | --- | --- | --- | --- | --- | --- | --- | --- | --- | --- | --- | --- | --- |
| (1) Ludyga 2020 | ✓ | NA | ✓ | ✓ | ✓ | NA | ✓ | ✓ | ? | ? | ✓ | 77.8% | Low |
| (2) Michel 2019 | x | NA | ✓ | ✓ | ✓ | NA | ✓ | ✓ | ✓ | ? | ✓ | 77.8% | Low |
| (3) Niederer 2011 | x | NA | ✓ | ✓ | ✓ | NA | ✓ | ✓ | ? | ? | ✓ | 66.7% | Moderate |
| (4) Rigoli 2013 | ✓ | NA | ✓ | ✓ | ✓ | NA | ✓ | ✓ | ✓ | NA | ✓ | 100.0% | Low |
| (5) Schmidt 2017 | x | NA | ✓ | ✓ | ✓ | NA | ? | ✓ | ? | NA | ✓ | 62.5% | Moderate |

Note: x = “no”, ✓= “yes”, ? = “unclear”, NA = “not applicable”.

**Figure S1.** The risk of bias assessment of the included studies (randomised controlled trials)


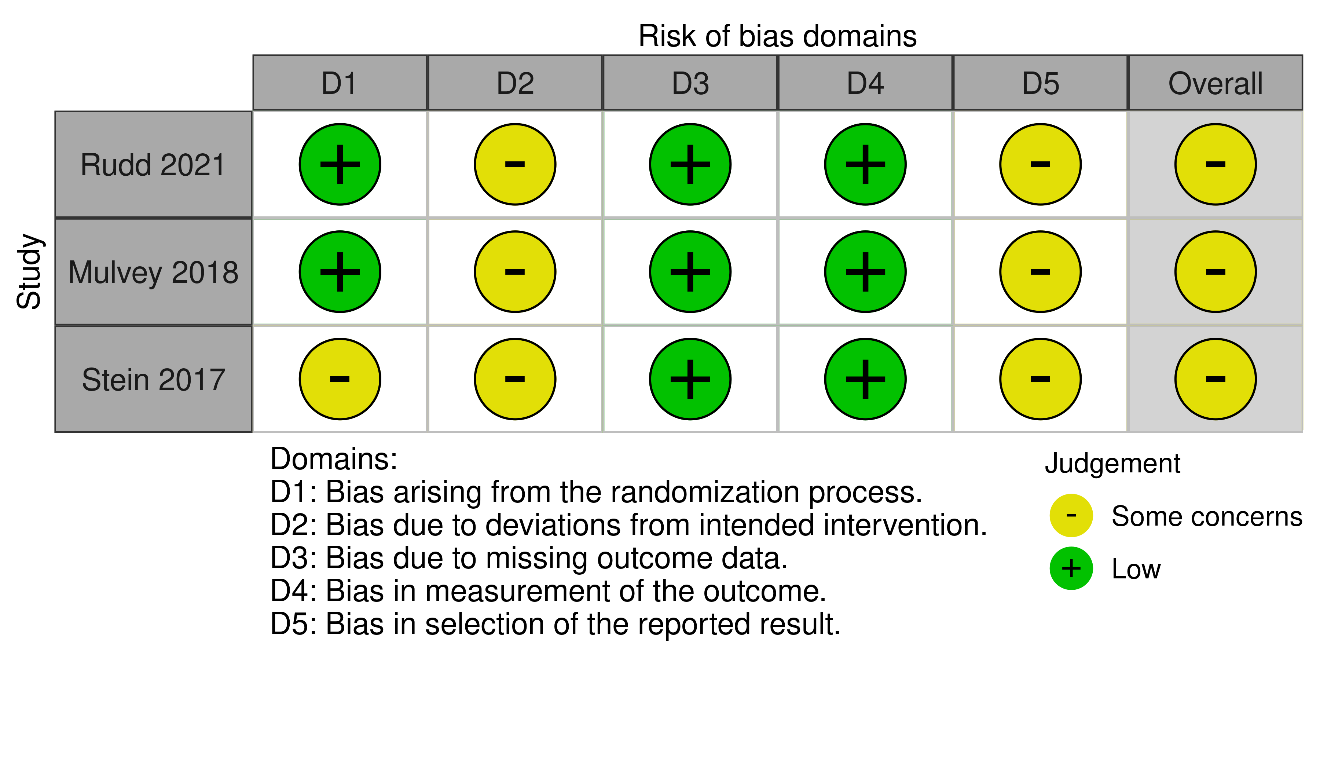


**Figure S2.** The risk of bias assessment of the included studies (non-randomised controlled trials)


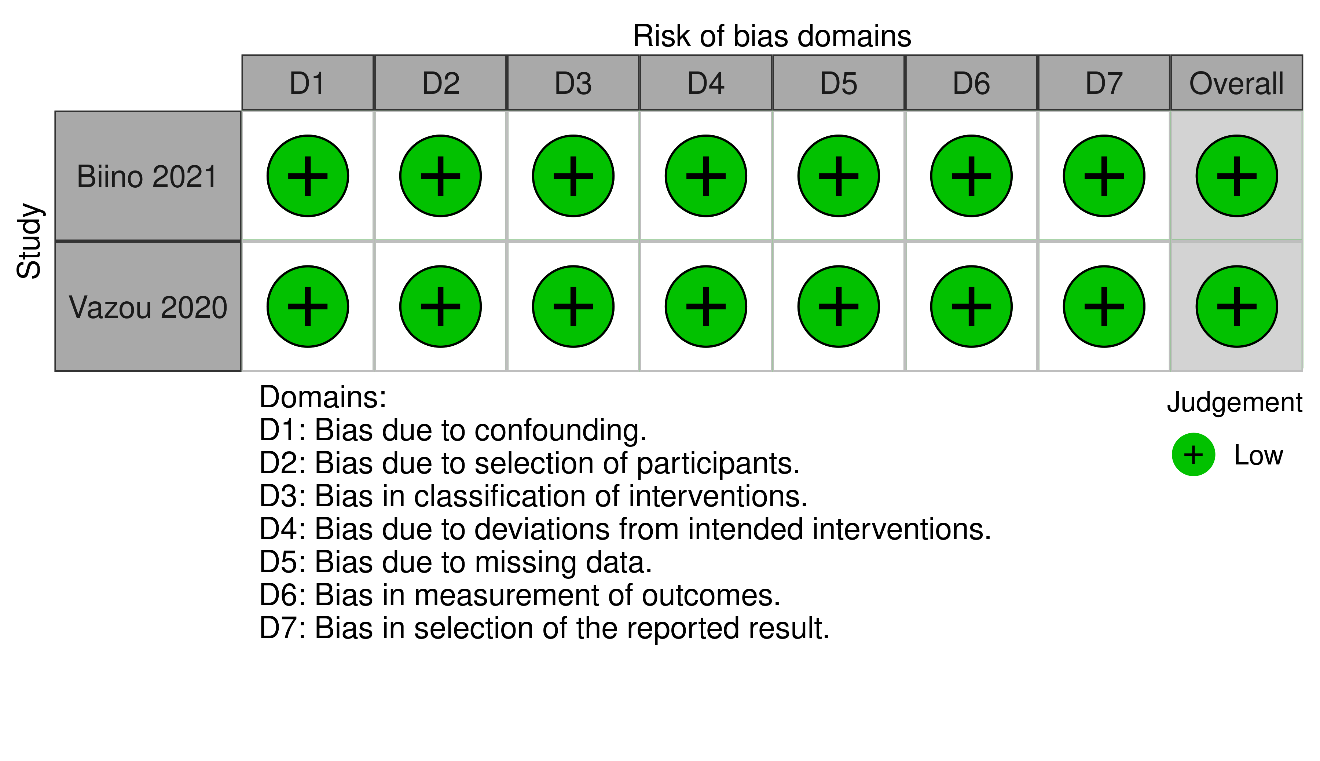

Supplement: Supplementary file 5 — Supplementary file5 (DOCX 442 KB) [file 40279_2024_2040_MOESM5_ESM.docx]
